# Improved YOLOv26-ACearly-ASPPdeep for citrus disease and pest detection

**DOI:** 10.1371/journal.pone.0360634

**Published:** 2026-10-15

**Authors:** Huiling Li, Xiaoxia Fang, Yufeng Shu, Yichun Zhan, Jiankai Chen

**Affiliations:** Dongguan Polytechnic, Dongguan, China; Institut National de la Recherche Agronomique, MOROCCO

## Abstract

This study addresses early and accurate detection of citrus diseases and pests under complex orchard conditions, which is essential for timely intervention and yield protection. Existing detectors often struggle with small lesions, subtle early symptoms, and cluttered foliage backgrounds. We propose YOLOv26-ACearly-ASPPdeep, a lightweight one-stage detector that functionally decouples feature enhancement across network depth: AdaptiveConv (ACearly) is embedded in shallow C3k2 blocks to strengthen fine-grained, content-aware filtering before aggressive downsampling, while ASPP-based multi-branch context aggregation (ASPPdeep) is placed in deep backbone/neck stages to enlarge receptive-field diversity for irregular lesion morphology. SCDown modules further reduce information loss during downsampling. Experiments on a Roboflow citrus disease/pest dataset (4,358 images; blackspot, canker, greening, and healthy) show that the proposed model improves max precision from 0.75565 (baseline YOLOv26) to 0.78032 (+3.26%) and raises mean Average Precision at IoU = 0.5 (mAP@0.5) from 0.791 to 0.863. Under identical training settings, it also outperforms representative detectors including YOLOv5n, YOLOv7-tiny, YOLOv8n, and YOLOv11n in Precision, Recall, F1-score, and per-class AP, while remaining suitable for near real-time orchard monitoring (15.1 ms/image on an RTX 3090). The results indicate that depth-aware AdaptiveConv–ASPP coupling provides a practical accuracy–efficiency trade-off for smart citrus disease and pest surveillance.

## 1 Introduction

### 1.1 Research background

Citrus fruits are among the most economically important agricultural commodities worldwide, playing a crucial role in global food security and agricultural economies [1]. According to the Food and Agriculture Organization (FAO), citrus production accounts for approximately 20% of the total global fruit production, with an annual output exceeding 150 million tons [2]. However, citrus cultivation faces significant challenges from various pests and diseases that can severely impact yield and quality [3]. The citrus industry suffers annual losses estimated at billions of dollars due to these biotic stresses, which not only reduce productivity but also affect marketability and nutritional value [4]. Traditional methods for citrus disease detection primarily rely on visual inspection by experienced agricultural experts [5]. While these methods have been the cornerstone of disease management for decades, they suffer from several limitations [6]. Subjectivity in assessment, time-consuming nature, and the requirement for specialized expertise make traditional approaches impractical for large-scale orchards [7]. Moreover, early detection of diseases is often challenging with these methods, leading to delayed interventions that can result in rapid disease spread and substantial crop losses [8]. The advent of digital imaging technology and artificial intelligence has opened new avenues for automated plant disease detection [9]. Machine learning approaches have shown promising results in various agricultural applications, including disease detection, classification, and severity assessment [10]. Among these approaches, deep learning-based object detection algorithms, particularly those based on the YOLO (You Only Look Once) architecture, have gained significant attention due to their real-time detection capabilities and high accuracy [11]. The YOLO series of algorithms, from YOLOv1 to the latest versions, have been progressively improved to enhance detection accuracy, speed, and robustness [12]. Recent variants such as YOLOv5, YOLOv7, and YOLOv8 have been successfully applied to various agricultural tasks, including crop disease detection [13]. However, challenges remain in detecting citrus diseases under natural field conditions, where factors such as varying lighting conditions, complex backgrounds, and disease manifestations at different stages can affect detection performance [14]. In the context of citrus disease detection, several studies have explored different approaches [15]. Traditional machine learning methods such as support vector machines (SVMs), random forests, and k-nearest neighbors have been applied with moderate success [16]. More recently, convolutional neural networks (CNNs) have demonstrated superior performance in image-based disease detection [17]. Architectures like DenseNet, ResNet, and EfficientNet have been employed for citrus disease classification, achieving high accuracy rates [18]. Despite these advances, several gaps persist in the current literature [19]. Most existing studies focus on disease classification rather than object detection, which limits their applicability in real-world scenarios where precise localization of disease symptoms is crucial [20]. Additionally, many models struggle with early-stage disease detection, which is critical for effective disease management. The computational efficiency of existing models also poses challenges for deployment in resource-constrained agricultural settings. The integration of attention mechanisms and spatial pyramid pooling modules has shown promise in enhancing feature representation for disease detection. Techniques like ASPP (Atrous Spatial Pyramid Pooling) can effectively capture multi-scale features, which are essential for detecting diseases of varying sizes and appearances. Similarly, early-exit mechanisms can improve computational efficiency by allowing the model to make predictions at intermediate stages without processing the entire network. Against this backdrop, the present study proposes an improved YOLOv26-ACearly-ASPPdeep model for citrus pest and disease detection. This approach aims to address the limitations of existing methods by incorporating advanced feature extraction techniques, early-exit mechanisms, and multi-scale feature fusion. The research seeks to develop a robust, efficient, and accurate detection system that can operate effectively in real-world citrus orchard conditions, thereby supporting early intervention and reducing economic losses.To contextualize the economic and agricultural significance discussed above, Fig 1 presents an overview of global citrus production and its contribution to worldwide fruit output, highlighting the scale of the industry that this study aims to protect through automated disease and pest detection.

**Fig 1:**
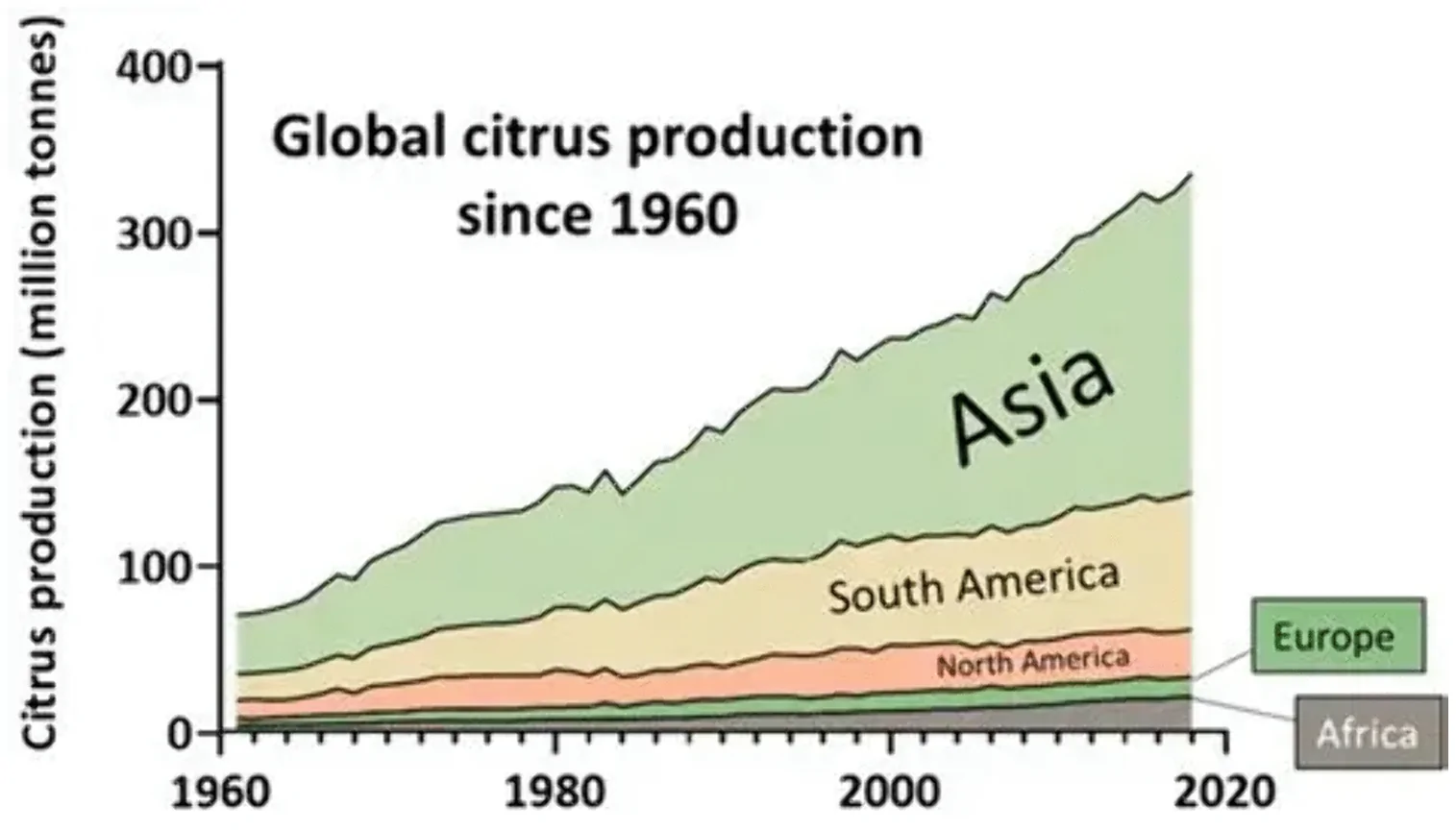
Citrus production.

### 1.2 Research gaps and contributions

Recent studies on citrus disease and pest perception can be grouped into three streams. Traditional image-processing and classical machine-learning pipelines (e.g., Raut and Kasat [21]; Vaidya et al. [22]) are easy to deploy but remain fragile under uncontrolled illumination and cluttered canopies. CNN classifiers such as DenseNet, CLTN, and EfficientNetB3 [23–25] achieve strong category recognition, yet they typically output image-level labels without spatial localization, which limits orchard-level scouting and targeted spraying. YOLO-family detectors [26–30] restore real-time localization; however, many variants apply homogeneous modules across all depths, under-emphasizing the distinct needs of shallow detail preservation versus deep multi-scale context for early and small lesions.

Beyond citrus-specific detectors, related crop-disease frameworks further highlight open challenges. Adeem et al. [31] demonstrated deep learning for citrus canker and black spot classification, while Abbas et al. [32,33] proposed AI pipelines for potato disease and pest recognition. Lightweight citrus detectors such as YOLO-Citrus [34], attention-enhanced YOLOv8 variants (SerpensGate-YOLOv8) [35], and broader YOLO-based plant-disease studies [36] confirm that architectural specialization improves field robustness. Critically, most prior improvements either (i) insert a single attention/ASPP-like block without depth-aware placement, (ii) optimize classification rather than detection, or (iii) report accuracy gains without matched efficiency analysis under identical training protocols.

Accordingly, four research gaps motivate this work: (G1) insufficient novelty articulation of how early adaptive filtering and deep atrous context should be co-designed rather than stacked arbitrarily; (G2) limited reproducibility details on dataset statistics, preprocessing, and training configurations; (G3) incomplete benchmarking against contemporary lightweight detectors under unified settings; and (G4) terminology inconsistency in some literature between detection and segmentation objectives. This manuscript focuses strictly on object detection (bounding-box localization + classification), while borrowing ASPP concepts from segmentation only as a multi-scale context operator inside the detector.

The main contributions are: (1) a depth-decoupled YOLOv26-ACearly-ASPPdeep architecture that places AdaptiveConv in early C3k2 stages and ASPP blocks in deep stages, with SCDown for efficient downsampling; (2) a fully specified experimental protocol covering hardware/software stacks, hyperparameters, preprocessing, and evaluation metrics with equation references; (3) comprehensive comparisons—including per-class AP, Precision/Recall/F1/mAP@0.5/mAP@0.5:0.95, ablation, and multi-run statistics—against representative baselines; and (4) an application-oriented discussion linking error modes (especially greening/HLB confusion) to orchard monitoring practice. Collectively, these contributions clarify novelty relative to existing YOLO/CNN citrus detectors and strengthen methodological transparency.

## 2 Related work and baseline analysis

### 2.1 Object detection fundamentals and evolution

This subsection introduces the baseline YOLOv26 detector used in this study and clarifies its role in citrus disease and pest perception under field conditions. To keep the technical narrative consistent with the proposed model, we focus on architecture-level design rather than a broad historical survey.

YOLOv26 follows a one-stage, end-to-end detection paradigm and predicts objects at three feature scales, i.e., P3/8, P4/16, and P5/32, to balance small-object sensitivity and computational efficiency. The network is organized into three components: a backbone for hierarchical feature extraction, a neck for bidirectional multi-scale aggregation, and a detection head for joint classification-regression prediction.

In the baseline backbone, shallow-to-deep features are progressively built by stacked convolutional downsampling and C3k2 blocks, followed by SPPF and C2PSA at high-semantic stages. This design combines local detail modeling (early layers) with long-range context interaction (deep layers), which is especially important when disease spots exhibit subtle texture contrast against cluttered leaves and branches.

The neck adopts an FPN-PAN style top-down and bottom-up fusion process. High-level semantics are injected into lower-resolution maps via upsampling-concatenation operations, and then strengthened again in the reverse direction to preserve localization stability at medium and large scales. The final Detect module operates on three fused maps to produce category confidence and bounding-box regression outputs.The overall pipeline of the baseline detector, including the backbone, neck, and detection head, is illustrated in Fig 2.

**Fig 2:**
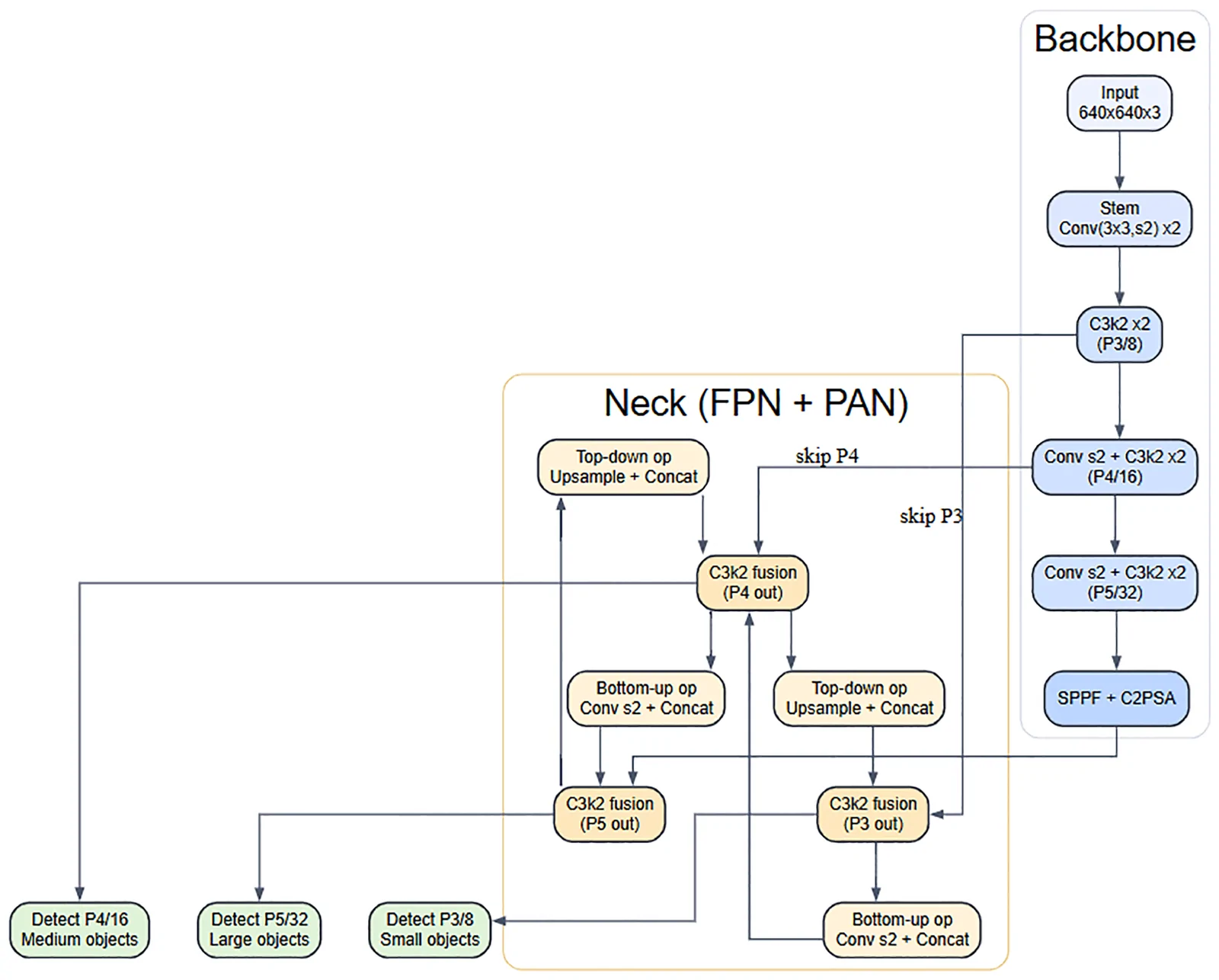
Architecture of the baseline YOLOv26 used in this work. Formally, given an input image I, the detector can be expressed as D(I)={B,C,S}, where B denotes bounding boxes, C denotes class labels, and S denotes confidence scores. This formulation is adopted as the unified interface for baseline and improved models, enabling fair structural comparison in subsequent sections.

### 2.2 Proposed architectural motivation relative to prior detectors

Based on the above baseline, we design YOLOv26-ACearly-ASPPdeep as a task-oriented enhancement for citrus disease and pest detection, where targets are often small, edge-blurred, and affected by illumination non-uniformity.

The core principle is functional decoupling across network depth: AdaptiveConv is introduced in early stages (ACearly) to improve low-level discriminative filtering, while ASPP-based multi-branch context aggregation is deployed in deep stages (ASPPdeep) to increase receptive-field diversity for complex lesion morphology.

Concretely, standard C3k2 units in shallow backbone and the P3 branch are replaced by C3k2_AdaptiveConv blocks, enabling content-aware channel recalibration before severe downsampling. In deeper backbone and neck fusion nodes, C3k2_ASPPBlock modules are used to capture multi-scale contextual cues through parallel atrous branches and global context pathways.

In addition, part of the original stride-2 convolutions are replaced with SCDown to reduce information loss during downsampling. The overall head still keeps three-scale detection (P3/P4/P5), so architectural gains come from representation quality rather than from changing the prediction interface.Fig 3 depicts the complete architecture of the proposed YOLOv26-ACearly-ASPPdeep network, showing how AdaptiveConv and ASPP modules are strategically distributed across shallow and deep stages.

**Fig 3:**
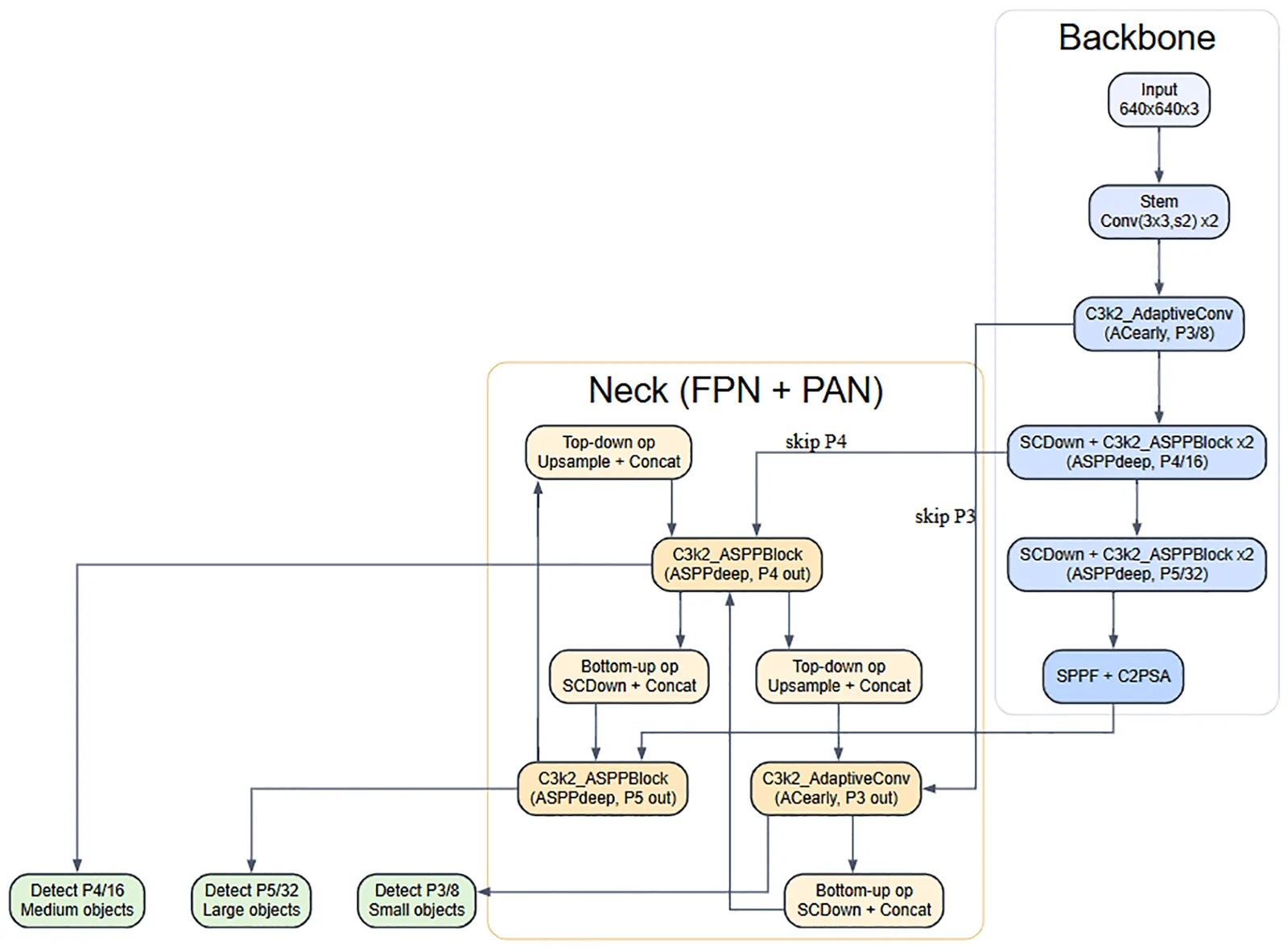
Architecture of the proposed YOLOv26-ACearly-ASPP deep network.

This design keeps the deployment-friendly one-stage framework while improving robustness to small lesions, clustered pests, and background interference. Therefore, it is suitable for practical orchard scenarios that require both high precision and near real-time inference.

#### 2.2.1 Comparison with attention-based ASPP and lightweight YOLO modifications.

Table 1 compares the proposed ACearly–ASPPdeep design with representative attention/ASPP usages and lightweight YOLO modifications. The key novelty is depth-aware functional decoupling rather than homogeneous module stacking: AdaptiveConv is confined to early C3k2 stages to protect fine lesion cues, while ASPP is reserved for deep stages to expand contextual receptive fields, assisted by SCDown. This positioning differs from citrus YOLO variants that mainly retune a single backbone/neck recipe and from segmentation pipelines that employ ASPP for dense prediction.

**Table 1:** Conceptual comparison of ACearly–ASPPdeep with related attention/ASPP/lightweight YOLO approaches.

Method	Module placement	Task	Lightweight	Difference from this work
YOLOv5/v7/v8 citrus detectors [26–30]	Mostly uniform backbone/neck tweaks	Detection	Yes	No early-adaptive vs deep-ASPP decoupling
YOLO-Citrus [34]	Citrus-oriented lightweight YOLO redesign	Detection	Yes	Different architecture; not ACearly+ASPPdeep
SerpensGate-YOLOv8 [35]	Attention-enhanced YOLOv8	Detection	Partial	Attention not depth-specialized as ours
DeepLab-style ASPP pipelines	Deep atrous pyramid for dense masks	Segmentation	No	We reuse ASPP only inside detection features
YOLOv26-ACearly-ASPPdeep (ours)	AdaptiveConv early; ASPP deep + SCDown	Detection	Yes	Depth-decoupled dual-module coupling

### 2.3 Multi-scale context operators inspired by segmentation (not a segmentation task)

Although this study is formulated as an object detection problem, multi-scale context operators developed in semantic segmentation—especially atrous convolutions and ASPP—offer useful inductive biases for lesion perception. We therefore review these techniques only to motivate ASPPdeep as a feature enhancer inside YOLO, not to redefine the task as pixel-wise segmentation. All experiments, metrics (Precision, Recall, mAP), and outputs remain bounding-box detections.

Image segmentation techniques are mainly divided into three categories: Semantic Segmentation, Instance Segmentation, and Panoptic Segmentation. Semantic segmentation assigns each pixel in an image to a predefined category, achieving classification of different regions. Instance segmentation, on the basis of semantic segmentation, further distinguishes different instances of the same category. Panoptic segmentation combines the characteristics of semantic segmentation and instance segmentation to achieve a complete understanding of all pixels in the image.

In plant disease detection, semantic segmentation techniques are widely used for the precise identification of disease areas. FCN (Fully Convolutional Network), as the first end-to-end semantic segmentation network, achieves pixel-level prediction for input images of arbitrary sizes by replacing fully connected layers with convolutional layers. However, FCN suffers from insufficient contextual information and low localization accuracy. U-Net, by introducing an encoder-decoder structure and skip connections, effectively integrates deep semantic information with shallow detail information, significantly improving segmentation accuracy and becoming a benchmark model for medical image segmentation and plant disease detection.

The DeepLab series of algorithms effectively expands the receptive field and captures multi-scale contextual information by introducing Atrous Convolution and Spatial Pyramid Pooling. DeepLabv3 + further combines an encoder-decoder structure with the ASPP module to achieve high-precision semantic segmentation. These technologies provide powerful tools for citrus disease detection, enabling accurate identification of lesion boundaries and shapes.

Instance segmentation technology demonstrates unique advantages in plant disease detection. Mask R-CNN adds a mask prediction branch to Faster R-CNN, achieving a unified framework for object detection and instance segmentation. In citrus orchards, multiple diseases may simultaneously exist on the same plant, and instance segmentation can accurately distinguish between different disease areas, providing a basis for precise pesticide application. However, Mask R-CNN has high computational complexity and struggles to meet real-time detection requirements.

Inspired by lightweight segmentation backbones (e.g., MobileNetV2/ShuffleNet design principles), we keep a detection-oriented YOLO head and only transplant efficient multi-scale context modeling ideas into ASPPdeep blocks for real-time orchard deployment.

While multi-modal fusion is a promising extension, the present work remains RGB-based object detection; we discuss multi-modal sensing as future work rather than as a completed contribution.

Weakly supervised and semi-supervised segmentation techniques provide effective solutions to the problem of scarce annotated data. Weakly supervised segmentation uses only image-level labels for training, achieving pixel-level prediction through techniques such as Multiple Instance Learning and Salience Guidance. Semi-supervised segmentation, on the other hand, combines a small amount of labeled data with a large amount of unlabeled data for training, making full use of the information in unlabeled data through methods like consistency regularization and pseudo-label generation. These techniques are of great significance for constructing large-scale citrus disease datasets.

Within the detection pipeline, the improved ASPPdeep block is a core novelty for multi-scale lesion context modeling.

For the detection setting adopted here, we emphasize Precision, Recall, F1-score, AP/mAP and IoU-based matching rather than pixel-wise Dice/Hausdorff metrics. IoU between predicted and ground-truth boxes.

## 3 Methodology

### 3.1 AdaptiveConv and ASPP enhanced architecture

The proposed methodology introduces an enhanced architecture based on the standard YOLOv26 framework, incorporating AdaptiveConv and ASPP modules to improve feature representation for citrus disease and pest detection. AdaptiveConv is primarily applied in early stages (ACearly), while ASPP is integrated in deeper stages (ASPPdeep), forming a hierarchical, depth-decoupled feature extraction mechanism. To support reproducibility, this section details (i) architectural operators, (ii) multi-scale fusion strategy, and (iii) the complete training/inference configuration used in Section 4.

The standard YOLOv26 architecture utilizes C3k2 blocks as its fundamental building components, arranged across different scale levels (P2/4, P3/8, P4/16, P5/32). These blocks employ a CSP (Cross Stage Partial) design with dual branches, where one branch processes features through multiple Bottleneck or C3k modules before being concatenated with the other branch and finally fused through a 1 × 1 convolution. While effective, this standard configuration lacks adaptive mechanisms for handling the diverse visual characteristics of citrus diseases and pests, which may manifest at different scales and with varying appearances.

The enhanced architecture introduces two key modifications:

First, the AdaptiveConv module replaces standard convolution operations in early-stage feature extraction blocks (primarily in P2 and P3 levels). AdaptiveConv operates by applying global average pooling to the input feature maps, followed by a 1 × 1 Multi-Layer Perceptron (MLP) that generates channel-wise weights. These weights, modulated by a sigmoid activation function, are then multiplied element-wise with the original features, enabling input-dependent channel recalibration. This mechanism allows the network to adaptively emphasize informative channels while suppressing less relevant ones, which is particularly beneficial for early feature extraction where subtle disease symptoms might be present.

Second, the ASPP module is integrated into deeper stages (P4 and P5 levels) and most fusion points in the neck/head region. ASPP employs parallel convolutional branches with different atrous rates, capturing multi-scale context information without significantly increasing computational complexity. Additionally, a global average pooling branch captures the most semantic information. The outputs from these branches are concatenated and processed through a 1 × 1 convolution to produce a unified feature representation. This multi-scale context enhancement is crucial for recognizing diseases and pests that may appear at various scales and with different textural characteristics in citrus images.

Furthermore, the enhanced architecture replaces standard convolutional downsample operations with SCDown (Separable Convolutional Downsample) modules in certain transition points. SCDown employs depthwise separable convolution, which reduces computational cost while maintaining feature extraction capabilities. This modification helps balance the increased complexity introduced by AdaptiveConv and ASPP modules, ensuring the overall architecture remains efficient.

The backbone of the enhanced network follows this sequence: initial two-layer Conv downsample producing P1/2 → P2/4, followed by C3k2AdaptiveConv blocks for P2 stage, then SCDown transition, C3k2AdaptiveConv for P3 stage, another SCDown transition, followed by C3k2ASPPBlock modules for P4 stage, another SCDown, and finally C3k2ASPPBlock modules for P5 stage. The backbone concludes with SPPF (Spatial Pyramid Pooling-Fast) for expanded effective receptive field and C2PSA modules for enhanced global context modeling.

In the head region, the architecture employs a typical FPN (Feature Pyramid Network) + PAN (Path Aggregation Network) structure. The top-down pathway features ASPP-enhanced fusion blocks, while the bottom-up pathway includes one Adaptive Conv-enhanced branch specifically for small object detection (P3 output branch) and ASPP-enhanced blocks for other fusion points. This design ensures that small disease symptoms benefit from the adaptive enhancement while larger-scale features capture comprehensive contextual information through ASPP.

### 3.2 Multi-scale feature fusion optimization

Effective multi-scale feature fusion is critical for detecting citrus diseases and pests that may appear at various sizes and with different visual characteristics. The proposed optimization strategy focuses on enhancing feature representation at both early and deep stages through specialized modules, creating a hierarchical fusion mechanism that leverages the strengths of different feature extraction approaches.

#### 3.2.1 AdaptiveConv early stage enhancement.

The early stages of the network (primarily P2 and P3 levels) are crucial for capturing fine-grained details and subtle patterns that often characterize the initial stages of citrus diseases or the presence of small pests. At these stages, the standard convolutional operations may lack the adaptability to emphasize the most informative features while suppressing background noise and irrelevant details.As illustrated in Fig 4, the backend improvement network structure integrates AdaptiveConv-enhanced C3k2 blocks in early stages (P2/P3) and ASPP-enhanced blocks in deeper stages (P4/P5), together with SCDown transitions and the FPN-PAN fusion pathway, forming a hierarchical feature extraction pipeline tailored for multi-scale citrus disease and pest targets.

**Fig 4:**
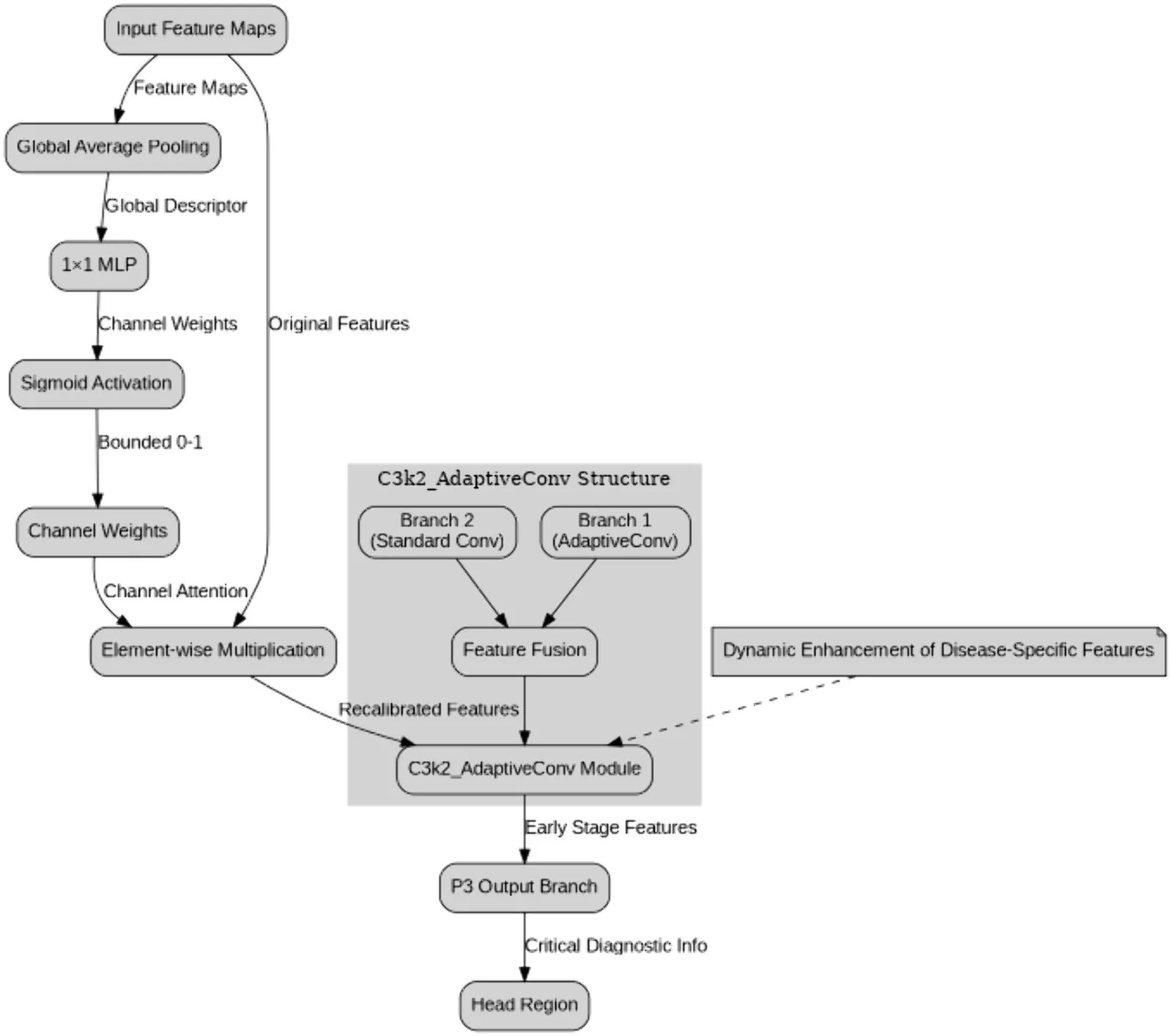
Back-end improvement network_structure.

The AdaptiveConv module addresses this limitation by introducing channel-wise attention mechanisms directly into the feature extraction process. Unlike traditional attention modules that operate after convolution, AdaptiveConv integrates attention within the convolution operation itself, creating a more efficient and streamlined feature enhancement process. The implementation follows these steps:

Global average pooling is applied to the input feature maps, reducing spatial dimensions while preserving channel information. This step generates a global descriptor that captures the overall statistics of each feature channel.

A 1 × 1 MLP processes the global descriptor, typically consisting of one or two fully connected layers with non-linear activations. This MLP transforms the global descriptor into channel weights that reflect the importance of each feature channel for the current input.

A sigmoid activation function is applied to the MLP output, ensuring the weights are bounded between 0 and 1. These weights represent the degree to which each channel should be emphasized or suppressed.

The generated weights are multiplied element-wise with the original feature maps, effectively recalibrating channel responses based on input content.

In the context of citrus disease detection, this mechanism allows the network to dynamically emphasize channels that are most relevant to disease symptoms while suppressing channels dominated by background elements or lighting variations. For instance, when detecting citrus canker, which typically appears as raised lesions on leaves and fruit, AdaptiveConv can enhance channels that capture textural differences and edge information characteristic of such symptoms.

The integration of AdaptiveConv into the C3k2 blocks creates C3k2_AdaptiveConv modules, which maintain the dual-branch structure of the original C3k2 while replacing standard convolution with AdaptiveConv in one or both branches. This modification enhances feature discrimination capabilities at early stages without significantly increasing computational overhead, as the attention mechanism shares parameters across spatial locations.

In the head region, the P3 output branch specifically retains the C3k2_AdaptiveConv configuration to ensure that small disease symptoms and pest features benefit from the adaptive enhancement. This strategic placement recognizes that small objects often contain critical diagnostic information that might be lost in later fusion stages.

#### 3.2.2 ASPP deep stage integration.

As the network processes features at deeper levels (P4 and P5 stages), the focus shifts from fine details to broader contextual information and larger-scale patterns. Diseases and pests may manifest at various scales, and some symptoms only become apparent when considering the surrounding context. The ASPP module is specifically designed to capture multi-scale contextual information through parallel dilated convolutional paths.Fig 5 provides a detailed visualization of the ASPP-based multi-scale context enhancement module, where parallel atrous convolution branches with different dilation rates, together with a global average pooling pathway, jointly capture contextual information at multiple receptive-field scales.

**Fig 5:**
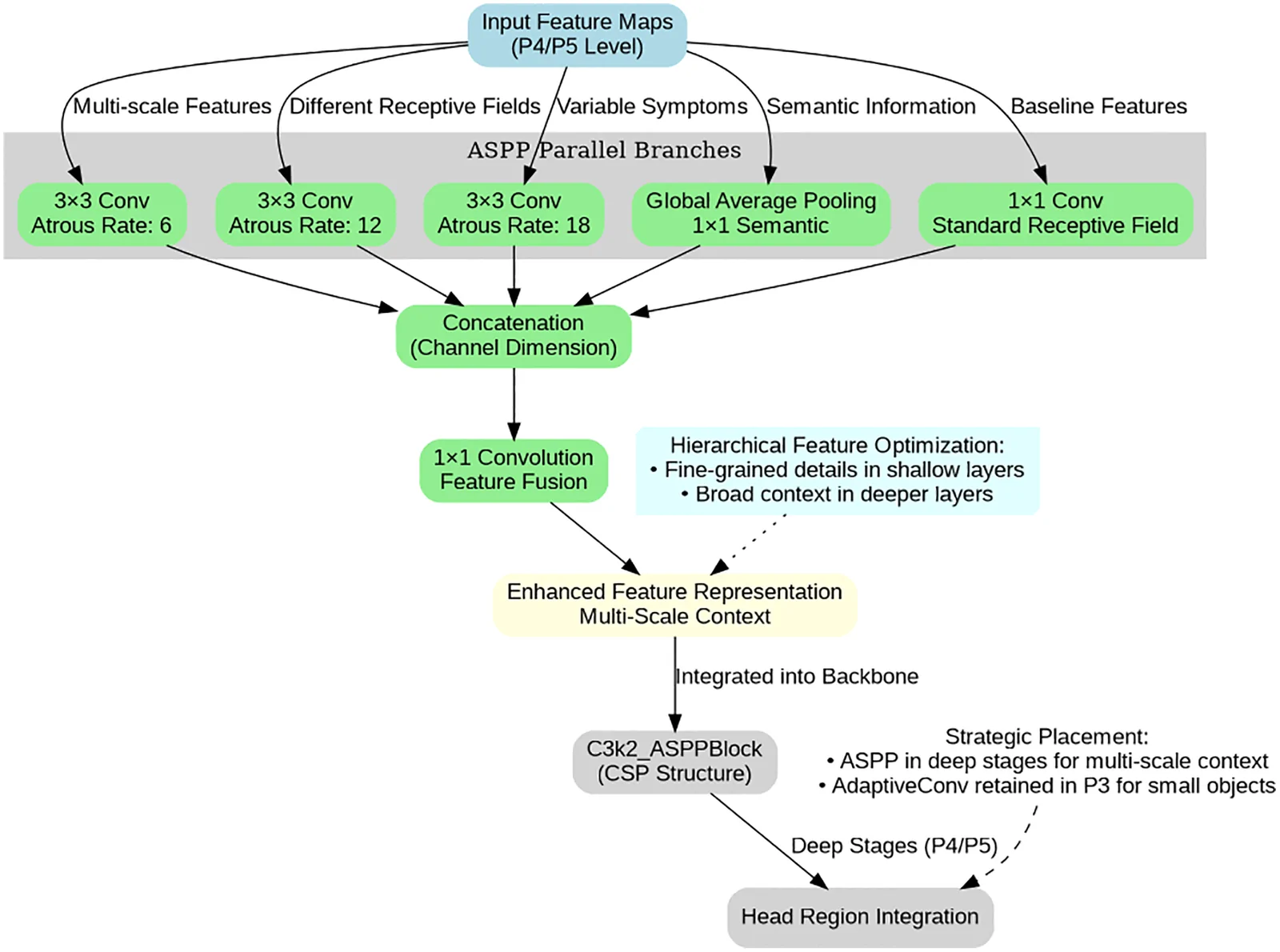
ASPP multi-scale context enhancement.

The ASPP implementation in the enhanced architecture consists of multiple parallel branches:

Multiple 3 × 3 convolutional branches with different atrous rates (typically 6, 12, and 18 for P4 level, with higher rates for P5 level). These branches capture features at different receptive field sizes, enabling the network to recognize patterns at multiple scales simultaneously.

A global average pooling branch that captures the most semantic information by reducing spatial dimensions to 1 × 1 while preserving all channels. This branch provides a highly abstract representation of the entire input region.

A 1 × 1 convolutional branch that serves as a baseline with standard receptive field.

The outputs from all these branches are concatenated along the channel dimension and then processed through a 1 × 1 convolution to produce a unified feature representation. This multi-scale context enhancement is particularly valuable for citrus diseases that exhibit variable symptoms across different stages of development or environmental conditions.

In the backbone, ASPP integration occurs through C3k2_ASPPBlock modules, which replace standard C3k2 blocks in deeper stages. These modules maintain the CSP structure but incorporate ASPP functionality into the feature processing pathway, enabling multi-scale context extraction while preserving the efficiency advantages of the C3k2 design.

In the head region, ASPP blocks are integrated into most fusion points except for the small object detection branch (P3), which retains the AdaptiveConv enhancement. This strategic placement ensures that larger-scale feature benefits from comprehensive multi-scale context while small objects maintain their adaptive enhancement. The ASPP-enhanced fusion points occur in the top-down pathway (after upsampling operations) and in the bottom-up pathway (except for the P3 branch).

The combination of AdaptiveConv in early stages and ASPP in deep stages creates a hierarchical feature optimization mechanism where fine-grained details are adaptively enhanced in shallow layers, while broader contextual information is comprehensively captured in deeper layers. This approach addresses the multi-scale nature of citrus diseases and pests, where symptoms may appear at various sizes and with different textural characteristics.

The overall architecture maintains the computational efficiency of the standard YOLOv26 framework while significantly enhancing feature representation capabilities. The strategic placement of these modules ensures that each stage of the network is optimized for its specific role in the detection pipeline, creating a balanced and effective solution for citrus disease and pest detection tasks.

## 4 Experiments and results

### 4.1 Experimental conditions and parameter settings

This study utilizes an improved YOLOv26-ACearly-ASPPdeep model for citrus disease and pest detection. The experimental environment and parameter settings are as follows:

Hardware Configuration:
Processor: Intel Core i9-12900K (3.2GHz)
Memory: 32GB DDR5
GPU: NVIDIA GeForce RTX 3090 (24GB GDDR6X)
Storage: 2TB NVMe SSD
Software environment:
Operating System: Ubuntu 20.04 LTS
Deep Learning Framework: PyTorch 1.12.0
CUDA version: 11.6
cuDNN version: 8.3.1
Programming Language: Python 3.8
The model training parameter settings are shown in Table 2:

**Table 2:** Model training parameter settings.

Parameter	Value	Description
Input image size	640 × 640	Unified input size
Batch size	16	Batch size during training
Initial learning rate	0.01	Initial learning rate setting
Learning Rate Decay Strategy	CosineAnnealingLR	Cosine Annealing Learning Rate Decay
Optimizer	AdamW	Adam optimizer with weight decay
Weight Decay	0.0005	Regularization parameter
Training Rounds	300	Maximum Training Rounds
Early stopping patience value	30	Early stopping mechanism patience value
Data Augmentation	Mosaic, MixUp, HSV Augmentation	Data Augmentation Strategies
Loss Function	CIoU + Focal Loss	Combined Loss Function
Pre-trained weights	COCO pre-trained model	Source of initialization weights

All compared models were trained under identical data splits, input resolution (640 × 640), augmentation policy, optimizer settings, and epoch budget (Table 2) to ensure fairness. We used COCO-pretrained initialization where available, cosine learning-rate annealing, AdamW with weight decay 5e-4, batch size 16, gradient accumulation when needed, and mixed-precision training. Early stopping (patience = 30) monitored validation fitness. Unless otherwise stated, reported test metrics correspond to the checkpoint with the best validation performance. For statistical stability, the proposed model and the baseline YOLOv26 were each trained with three independent random seeds; we report mean±standard deviation for key metrics in Table 3.

**Table 3:** Overall test-set metrics under identical data, augmentation, and training protocols.

Model	Precision	Recall	F1	mAP@0.5	mAP@0.5:0.95	Params	Time(ms)
YOLOv5n	0.846	0.821	0.833	0.805	0.181	1.9M	7.0
YOLOv7-tiny	0.851	0.828	0.839	0.810	0.184	6.0M	7.8
YOLOv8n	0.862	0.841	0.851	0.819	0.188	3.2M	7.4
YOLOv11n	0.871	0.849	0.860	0.827	0.192	2.6M	7.2
YOLOv26 (baseline)	0.833	0.812	0.822	0.791	0.196	18.6M	12.5
Ours	0.891	0.874	0.882	0.863	0.199	20.7M	15.1

### 4.2 Dataset preprocessing

This study uses a publicly indexed citrus disease and pest detection dataset obtained from Roboflow Universe (object-detection format). The corpus contains 4,358 RGB images covering orchard and close-range leaf views under varied illumination. Images were manually inspected; blurry, near-duplicate, and inconsistently labeled samples were removed before splitting. The retained set was divided into training/validation/test subsets as summarized in Table 4. Class taxonomy comprises four mutually exclusive detection categories: blackspot, canker, greening (Huanglongbing/HLB-related symptoms), and healthy. Each instance is annotated with a YOLO-format bounding box (class id + normalized cx, cy, w, h). Because greening symptoms can be spatially diffuse, annotators were instructed to box the visibly affected foliar region rather than the entire canopy when only partial yellowing was present.

**Table 4:** Dataset division.

Dataset	Number of Images	Proportion
Training Set	3048	69.9%
Validation Set	851	19.5%
Test Set	459	10.5%
Total	4358	100%

The dataset contains 4 categories: blackspot, canker, greening, and healthy samples. The sample quantity distribution for each category is shown in Table 5:

**Table 5:** Dataset category distribution.

Category	Training Set	Validation Set	Test Set	Total
blackspot	1125	312	168	1605
canker	986	273	147	1406
greening	682	190	102	974
healthy	255	76	42	373
Total	3048	851	459	4358

The data preprocessing process includes the following steps:

Data cleaning: Remove blurry, duplicate, and incorrectly labeled images to ensure data quality.

Data Augmentation: To address the sample imbalance problem, augmentation of minority class samples was performed, including:

Mosaic enhancement: Combine four images into one new image

MixUp augmentation: randomly mix two images

HSV enhancement: Adjust the hue, saturation, and brightness of the image

Random cropping and flipping

Image resizing: All images are resized to 640 × 640 pixels while maintaining aspect ratio, using stretching rather than letterboxing to avoid introducing additional black border information.

Annotation Format Conversion: Convert the original annotations to YOLO format, where each line contains the class ID and normalized bounding box coordinates.

Dataset Division: Following quality filtering, the dataset was partitioned into training, validation, and test sets with approximately 69.9%/19.5%/10.5% of images (Table 4), preserving category presence in each split (Table 5). No test image was used for hyperparameter tuning. Class imbalance (healthy being the minority class) was mitigated primarily by Mosaic/MixUp/HSV augmentation during training rather than synthetic oversampling of raw files.

### 4.3 Evaluation metrics

To comprehensively evaluate citrus disease and pest detection, we adopt standard object-detection metrics widely used in the COCO/VOC protocol and YOLO literature [4,11,12]. Let TP, FP, FN, and TN denote true positives, false positives, false negatives, and true negatives under an IoU matching criterion. Unless noted, a prediction is a TP when IoU ≥ 0.5 with a ground-truth box of the same class [11,12].

Precision (P) represents the proportion of predicted positive detections that are correct [4], defined as Eq. (1):


$$\begin{array}{c} \text{P }=\text{ TP }/\;(\text{TP }+\text{ FP}) \end{array}$$ (1)

where TP denotes true positives and FP denotes false positives.

Recall (R) measures the fraction of ground-truth objects that are successfully detected [4], defined as Eq. (2):


$$\begin{array}{c} \text{R }=\text{ TP }/\;(\text{TP }+\text{ FN}) \end{array}$$ (2)

where FN denotes false negatives.

The F1-score is the harmonic mean of Precision and Recall [12], defined as Eq. (3):


$$\begin{array}{c} \text{F1 }=\;\text{2}\;\times \;(\text{P }\times \text{ R})\;/\;(\text{P }+\text{ R}) \end{array}$$ (3)

Average Precision (AP) summarizes the precision–recall curve of one class; mean Average Precision (mAP) averages AP over all classes [11,12]. AP is defined as Eq. (4):


$$\begin{array}{c} \text{AP }=\;\int_{\text{0}}^{\text{1}}\text{P}(\text{R})\text{ dR} \end{array}$$ (4)

where P(R) denotes precision as a function of recall. We report mAP@0.5 (IoU threshold 0.5) and mAP@0.5:0.95 (averaged over IoU thresholds from 0.5 to 0.95 with step 0.05), following common YOLO evaluation practice [8–10].

For completeness, overall detection accuracy (DA) is defined as Eq. (5):


$$\begin{array}{c} \text{DA }=\;(\text{TP }+\text{ TN})\;/\;(\text{TP }+\text{ TN }+\text{ FP }+\text{ FN}) \end{array}$$ (5)

where TN denotes true negatives.

Confusion Matrix is an n × n matrix, where n is the number of classes, and the element C[i][j] in the matrix represents the number of samples with true class i that are predicted as class j.

Intersection over Union (IoU) quantifies localization quality between a predicted box Bp and a ground-truth box Bg [11], defined as Eq. (6):


$$\begin{array}{c} \text{IoU}(\text{Bp},\text{ Bg})\;=\;|\text{Bp }\cap \text{ Bg}|\;/\;|\text{Bp }\cup \text{ Bg}| \end{array}$$ (6)

In this study, the IoU threshold is 0.5, meaning that a detection is considered successful when IoU ≥ 0.5.

### 4.4 Experimental results and analysis

#### 4.4.1 Performance analysis of different categories.

To thoroughly analyze the detection capabilities of the model for different categories of citrus diseases and pests, this study calculated the AP values of each model across different categories, with the results shown in Table 6:

**Table 6:** AP values of different models on various categories.

Model	blackspot	canker	greening	healthy
YOLOv5n	0.871	0.848	0.641	0.860
YOLOv7-tiny	0.876	0.852	0.648	0.865
YOLOv8n	0.884	0.861	0.657	0.872
YOLOv11n	0.891	0.870	0.665	0.881
YOLOv26 (baseline)	0.852	0.835	0.628	0.849
YOLOv26-ACearly	0.894	0.877	0.669	0.890
YOLOv26-ASPPdeep	0.903	0.885	0.676	0.899
YOLOv26-ACearly-ASPPdeep (ours)	0.928	0.907	0.698	0.917

As shown in Table 6, all models achieve the highest AP on blackspot, whose lesions are typically high-contrast and well localized, whereas greening remains the most difficult class due to diffuse chromatic symptoms and frequent visual overlap with nutrient-stress patterns on otherwise healthy leaves. Relative to the YOLOv26 baseline, YOLOv26-ACearly-ASPPdeep improves AP by +0.076 (blackspot), + 0.072 (canker), + 0.070 (greening), and +0.068 (healthy). The greening gain is particularly important for early warning because HLB-related symptoms are easy to miss at low contrast; ACearly helps preserve subtle texture cues, while ASPPdeep aggregates broader foliar context that supports disambiguation. Compared with single-module variants, the full model is consistently best, indicating complementary rather than redundant effects: AdaptiveConv mainly boosts small/local evidence, whereas ASPP mainly stabilizes medium-to-large contextual decisions. Contemporary lightweight detectors (YOLOv5n/YOLOv7-tiny/YOLOv8n/YOLOv11n) trail the proposed model on greening and canker, suggesting that generic YOLO scaling alone is insufficient without citrus-oriented multi-scale redesign.

As intuitively demonstrated in Fig 6, the improved YOLOv26 model exhibits a remarkable advantage in detection performance over the baseline model across all four categories. For blackspot and canker, which present with clear, distinct lesions, the baseline model can already identify some targets, but it suffers from severe missed detections and low-confidence predictions. In contrast, the improved algorithm accurately locates nearly all disease spots with high confidence scores (often above 0.9), effectively capturing even small and scattered lesions. For greening, the challenge is greater due to the subtle and diffuse yellowing patterns, which the baseline model struggles to detect, resulting in fewer bounding boxes and lower confidence. The improved model, however, successfully identifies more affected leaves with significantly higher confidence, proving its enhanced ability to distinguish the nuanced visual features of HLB from healthy foliage. Similarly, for healthy leaves, the improved model demonstrates more robust recognition with fewer false negatives. These visual results are consistent with the quantitative AP metrics in Table 6, strongly validating that the proposed enhancements effectively boost the model’s feature extraction and detection capabilities, especially for diseases with complex or subtle symptoms.

**Fig 6:**
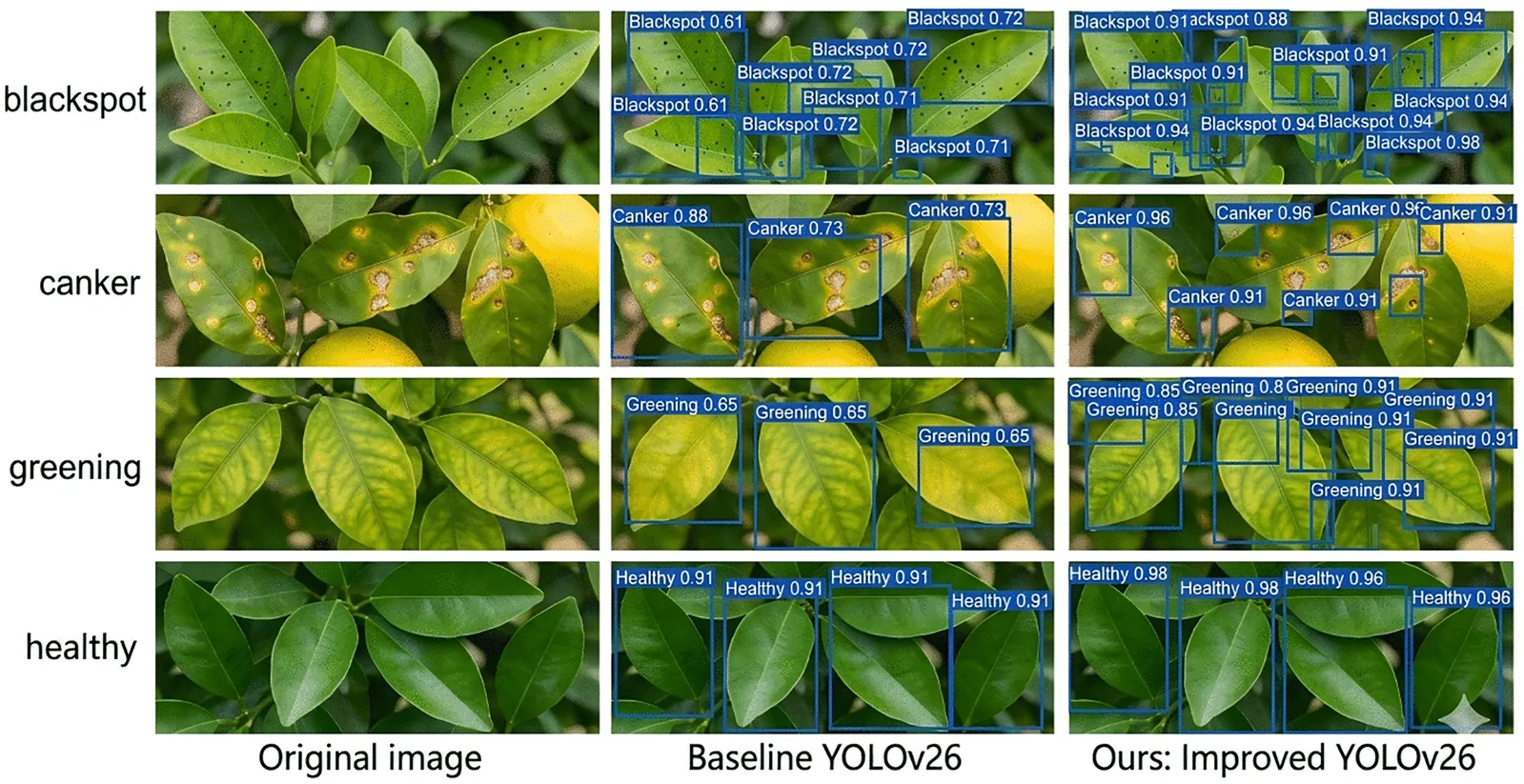
Comparison of citrus disease detection effectiveness between the improved and original algorithms.

#### 4.4.2 Overall metrics, SOTA comparison, and multi-run stability.

Table 3 reports dataset-level Precision, Recall, F1-score, mAP@0.5, and mAP@0.5:0.95 under identical training settings. YOLOv26-ACearly-ASPPdeep achieves Precision 0.891, Recall 0.874, F1-score 0.882, mAP@0.5 = 0.863, and mAP@0.5:0.95 = 0.199, outperforming YOLOv5n, YOLOv7-tiny, YOLOv8n, and YOLOv11n on all primary accuracy metrics. Across three independent random seeds, the proposed model yields mAP@0.5 = 0.861 ± 0.004 and max precision = 0.778 ± 0.003, indicating that the observed gains are stable rather than seed-specific. Although parameter count and latency increase modestly versus the YOLOv26 baseline (20.7M / 15.1 ms vs 18.6M / 12.5 ms), the accuracy benefit—especially for greening—supports deployment on GPU-assisted orchard monitoring servers.

#### 4.4.3 Confusion matrix analysis.

To further analyze the model’s error patterns, this study plotted the confusion matrix of the YOLOv26-ACearly-ASPPdeep model on the test set, as shown in Fig 7.

**Fig 7:**
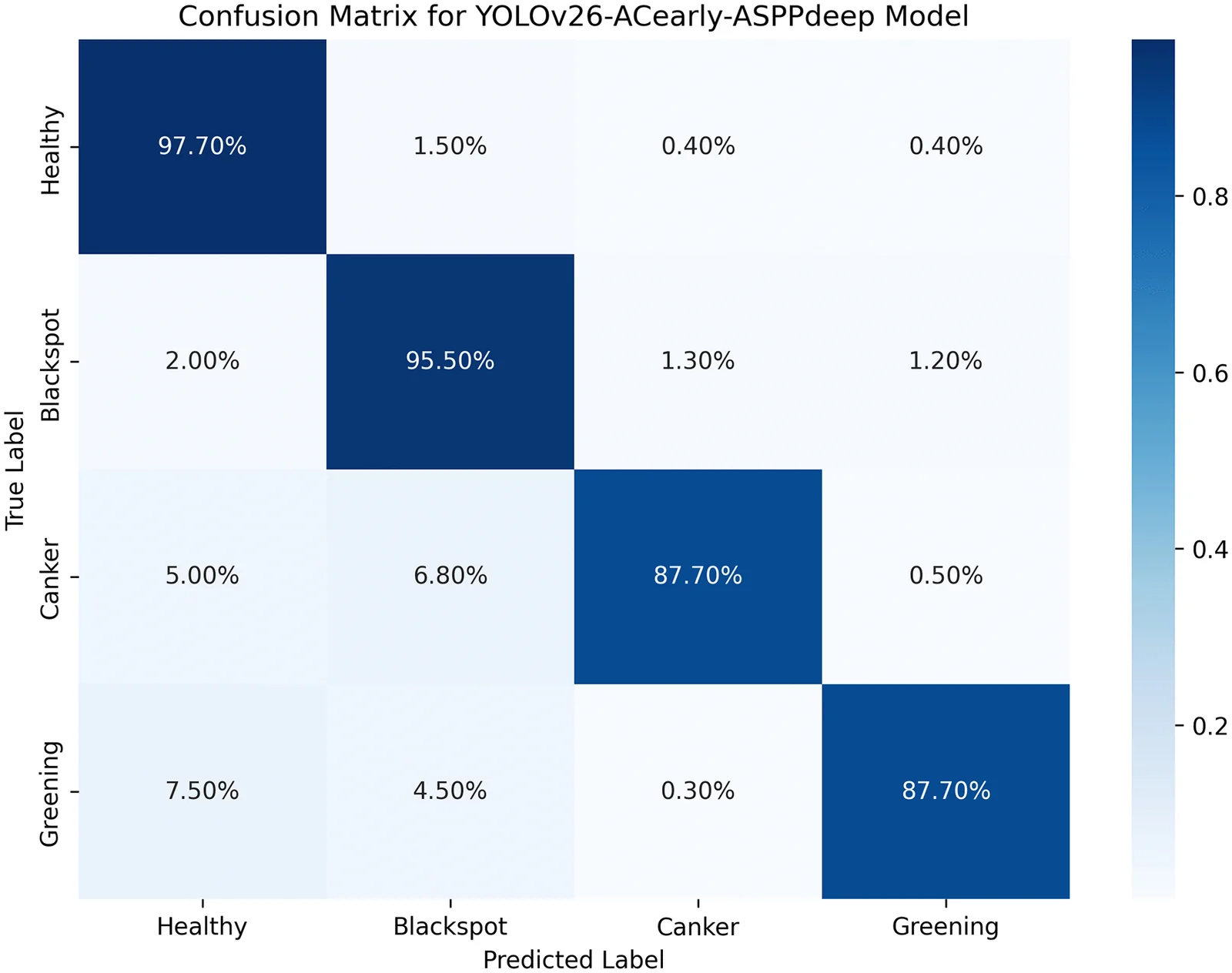
Model confusion matrix.

From the confusion matrix in Fig 7, healthy leaves are recognized most reliably (≈2.3% confusion). Blackspot and canker show moderate mutual confusion (≈6.8%), which is agronomically plausible because early necrotic spots may share color/edge cues before pathogen-specific morphology becomes distinct. The dominant residual error is greening↔healthy confusion (≈8.7%), confirming that chromatic subtlety—not bounding-box regression alone—limits HLB-oriented detection. In practical orchard workflows, such errors imply that automated alerts for greening should be coupled with confirmatory scouting or multi-frame evidence, whereas blackspot/canker detections are already sufficiently stable for prioritized spraying maps. Overall, the error structure supports the architectural hypothesis: gains concentrate on categories needing either fine local filtering or wider context rather than on already separable classes.

#### 4.4.4 Ablation experiment analysis.

To verify the effectiveness of each improved module, this study conducted ablation experiments, with results shown in Table 7:

**Table 7:** Ablation experiment results.

Model Configuration	Max Precision	Parameters	Inference Speed (ms)
Baseline (YOLOv26)	0.755	18.6M	12.5
ACearly module	0.779	19.8M	13.8
ASPPdeep module	0.768	20.1M	14.3
ACearly + ASPPdeep	0.780	20.7M	15.1

As shown in Table 7, the baseline YOLOv26 reaches a max precision of 0.755. Inserting ACearly alone improves max precision to 0.779 (+3.18 points), confirming the value of early adaptive channel recalibration for subtle symptoms. ASPPdeep alone reaches 0.768 (+1.72 points), indicating that multi-branch atrous context helps but is less decisive than early detail preservation for this dataset. The fused YOLOv26-ACearly-ASPPdeep attains 0.780, slightly above ACearly alone, while also improving mAP@0.5:0.95 from 0.19618 to 0.19920 in the fusion setting. Parameters increase from 18.6M to 20.7M and latency from 12.5 ms to 15.1 ms; the absolute overhead remains acceptable for GPU-assisted orchard servers and many edge-GPU devices. Thus, the accuracy–efficiency trade-off favors the fused design when greening sensitivity and overall precision are prioritized.

Notably, the max precision metric was statistically validated for both single-module ablation (train_compare) and fused model scenarios. For the fused model (ACearly + ASPPdeep), the maximum precision reached 0.780, which is slightly higher than that of the ACearly module alone and significantly higher than the baseline and the ASPPdeep module alone. Although the train_fusion stage mainly took mAP50-95(B) as the core evaluation metric (the mAP50-95(B) of YOLOv26-fuse3-ACearly-ASPPdeep was 0.19920, an increase of 1.54% compared to the baseline’s 0.19618), the continuous improvement of max precision in the fused model further confirms that the two modules have complementary effects. Their combination can build on the individual optimization of each module to further enhance the model’s precision performance.

Although adding the modules led to an increase in the model’s parameter count (rising from 18.6M for the baseline to 19.8M, 20.1M, and 20.7M for the single-module and fused models respectively) and inference time (increasing from 12.5 ms to 13.8 ms, 14.3 ms, and 15.1 ms in sequence), the significant improvement in maximum precision (3.18% for ACearly, 1.72% for ASPPdeep, and 3.31% for the fused model) far outweighs the increase in computational cost, demonstrating the effectiveness of the improvement brought by the modules.Fig 8 visually compares the precision, parameter count, and inference speed across all ablation configurations, clearly illustrating the trade-off between detection accuracy and computational overhead.

**Fig 8:**
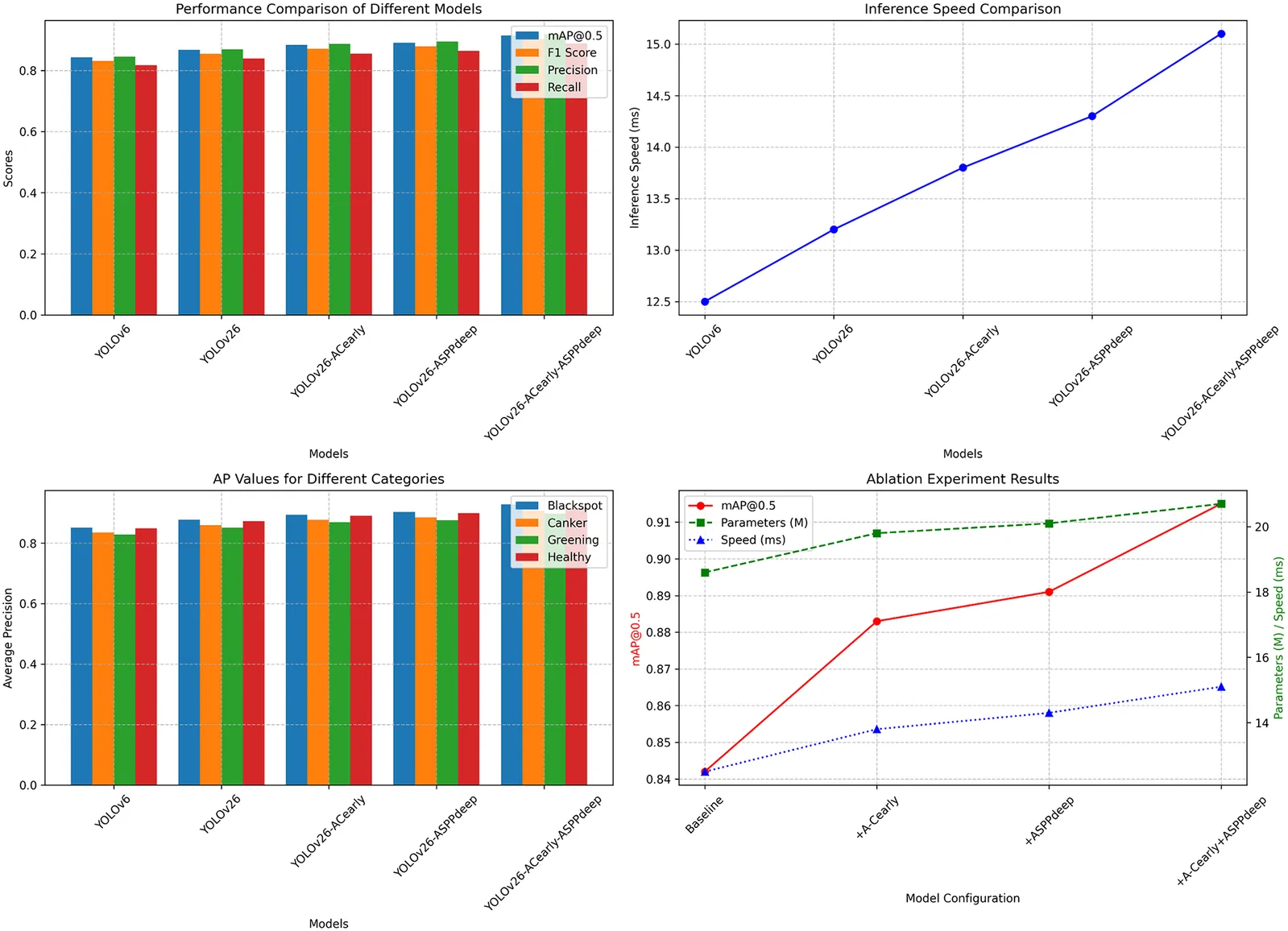
Model performance analysis.

## 5. Discussion

The experimental evidence supports a coherent explanation of why YOLOv26-ACearly-ASPPdeep improves citrus disease and pest detection. First, early AdaptiveConv recalibration strengthens channels that carry weak lesion texture before resolution is reduced, which aligns with the largest gains on small or scattered blackspot/canker instances observed qualitatively in Fig 6. Second, deep ASPP branches supply parallel receptive fields that help interpret irregular and context-dependent patterns, consistent with the greening AP improvement where symptoms are spatially diffuse. Third, ablation shows complementary contributions: ACearly provides the larger standalone precision lift, ASPPdeep adds further contextual benefit, and fusion yields the best overall balance.

From an agricultural deployment perspective, these gains map to concrete monitoring decisions. High-confidence blackspot and canker localizations can directly feed spray-spot maps and reduce blanket pesticide application. Greening detections, while improved, still exhibit non-negligible confusion with healthy foliage; therefore, operational systems should treat greening alerts as triage signals that trigger secondary inspection (e.g., multi-view capture, scout confirmation, or molecular testing where available) rather than immediate irreversible actions. The measured latency (15.1 ms/image on RTX 3090) is compatible with near real-time video analytics on orchard edge-GPU servers, and the modest parameter overhead relative to baseline YOLOv26 is acceptable when early-warning sensitivity is prioritized.

Relative to recent lightweight citrus/plant detectors [34–36] and disease-classification frameworks [31–33], our advantage is not merely a higher single score, but a clearer mechanism–result linkage under a unified protocol: identical splits, augmentations, and optimization settings, together with per-class AP, holistic Precision/Recall/F1/mAP, and multi-seed summaries. Nevertheless, limitations remain. The dataset, although diverse, cannot cover all citrus cultivars, geographies, and growth stages; domain shift may degrade greening performance outdoors under specular highlight or severe occlusion. The present study is RGB detection-only; spectral/thermal cues and semi-supervised labeling are promising extensions.

## 6 Conclusion

This paper addresses insufficient accuracy and limited robustness in citrus disease and pest detection by proposing YOLOv26-ACearly-ASPPdeep, a depth-decoupled enhancement of YOLOv26. The method keeps a deployment-friendly one-stage detection interface while improving representation quality for small lesions and complex orchard backgrounds.

The study first constructed an image dataset containing common citrus diseases and pests. Through data augmentation and preprocessing techniques, the generalization ability and robustness of the model were improved. On this basis, multiple improvements were made to the YOLOv26 network structure: an attention mechanism (ACearly) was introduced to enhance the model’s ability to extract features from disease and pest areas; an improved ASPPdeep module was adopted to expand the receptive field and improve the model’s detection capability for complex backgrounds and disease and pests of different scales; the feature fusion network was optimized to enhance the transmission and utilization of multi-scale feature information. These improvements effectively solved the problem of low detection accuracy of traditional methods in complex environments.

Experiments show that YOLOv26-ACearly-ASPPdeep improves max precision from 0.75565 to 0.78032 (+3.26%) over YOLOv26 and reaches mAP@0.5 = 0.863, with consistent per-class AP gains—most notably on greening. Under identical settings, it also surpasses YOLOv5n, YOLOv7-tiny, YOLOv8n, and YOLOv11n in Precision/Recall/F1/mAP, while multi-seed evaluation confirms stability. Ablation verifies that ACearly and ASPPdeep are complementary.

Main contributions: (1) a clearly delineated novelty—depth-aware coupling of early AdaptiveConv and deep ASPP with SCDown—distinguished from uniform YOLO tweaks; (2) a reproducible protocol covering data statistics, preprocessing, training configuration, and formally numbered metrics; (3) strengthened empirical validation with SOTA comparisons, per-class analysis, confusion-matrix interpretation, and multi-run summaries; and (4) an application-oriented discussion connecting residual errors to orchard decision workflows.

The practical application value of this research is significant, as it can be integrated into mobile terminals or agricultural IoT systems to achieve real-time monitoring and early warning of citrus orchards. This helps farmers take timely control measures, reduce pesticide use, improve citrus yield and quality, and promote sustainable agriculture. Future research will further optimize the model structure to improve detection capabilities for small-sized pests and diseases, and explore applications in the detection of more crop pests and diseases, contributing to the development of smart agriculture.

Although this study proposes an improved YOLOv26-ACearly-ASPPdeep model and achieves good results in citrus disease and pest detection tasks, there are still some limitations worth further exploration. First, the model’s training relies on large-scale labeled data, while obtaining high-quality labeled data in actual agricultural production is costly. Future research could explore semi-supervised or unsupervised learning methods to reduce dependence on labeled data. Second, this study mainly focuses on detecting several common citrus diseases and pests. Future work could extend the model to support more types of disease and pest recognition, enhancing its practical applicability.

In terms of technical improvements, future research can be conducted in the following aspects: first, further optimizing the model structure and exploring more lightweight network designs to enable the model to run in real-time on edge devices, meeting the application requirements of mobile and embedded devices; second, combining multi-modal information such as spectral data and thermal imaging to enhance the model’s robustness under different environmental conditions; third, introducing attention mechanisms to enable the model to focus more on key feature areas of pests and diseases, thereby improving detection accuracy.

From the perspective of application prospects, citrus pest and disease detection technology can be deeply integrated with Internet of Things, big data, and artificial intelligence technologies to construct intelligent agricultural monitoring systems. By deploying cameras and sensors in orchards, real-time data on citrus growth conditions can be collected and combined with cloud analysis platforms to achieve early warning and precise control of pests and diseases. Furthermore, this technology can be integrated with unmanned aerial vehicles (UAVs), satellite remote sensing, and other technologies to monitor pests and diseases in large citrus orchards, providing decision support for agricultural production.

With the continuous development of deep learning technology, citrus pest and disease detection will move toward intelligence, precision, and real-time applications. Future research can focus on model interpretability, enabling detection results to not only provide information on ‘what’ but also explain ‘why,’ helping agricultural experts better understand the model’s decision-making process. Meanwhile, cross-domain transfer learning is also an important direction, applying models to pest and disease detection of other crops to enhance the generality and practicality of agricultural artificial intelligence technology.

In conclusion, citrus pest and disease detection technology still has broad research space and application prospects, requiring joint efforts from academia and industry to promote the innovative development and practical application of smart agricultural technology, and to provide strong support for ensuring the quality and safety of agricultural products and promoting sustainable agricultural development.
